# Individual differences in social intelligence and perception of emotion expression of masked and unmasked faces

**DOI:** 10.1186/s41235-022-00408-3

**Published:** 2022-06-28

**Authors:** Riley H. Swain, Aminda J. O’Hare, Kamila Brandley, A. Tye Gardner

**Affiliations:** 1 Department of Psychological Science, Weber State University, Ogden, UT USA; 2 Department of Electrical and Computer Engineering, Weber State University, Ogden, UT USA

**Keywords:** Face masks, Emotion perception, Face perception, Social intelligence

## Abstract

Facial expressions provide key information for successful social interactions. Recent research finds that accurate perception of emotion expressions decreases when faces are presented with face masks. What is unknown is how individual differences in social intelligence may influence perception of masked emotion expressions. In this study, participants (*n* = 224) completed an emotion perception task of face stimuli presented with and without face masks and completed two measures of social intelligence: the Reading the Mind in the Eyes Test (RMET) and the Tromsø Social Intelligence Scale (TSIS). Face masks were found to significantly decrease the accurate identification of emotion expressions, impacting the perception of disgust and sad expressions the most. Further, the type of emotion misattributed to facial expressions varied across expressions. Performance on the RMET test did predict perception accuracy, while scores on the TSIS did not. As face masks continue to be common globally, we must be aware that they cause interference with our social interactions and perceptions. Further, some individuals may be more negatively impacted by these effects than others. As such, it is important that we find ways to ensure that we are effectively communicating with one another and have patience when perception mistakes arise.

## Significance statement

Our study supports findings that the use of face masks can interfere with our ability to accurately detect the emotion expressions of others. While this effect seems to impact the majority of individuals studied, not all individuals are impacted to the same degree. We find that as individuals increase in social intelligence, their emotion detection accuracy also increases. As such, individuals who struggle with social intelligence are likely experiencing even more miscommunications and misattribution of emotion when face masks are in use. It is important to be cognizant of these deficits, while face masks are in use for successful social interactions.

## Introduction

Facial recognition is important for social interactions, and the emotional perception of these interactions can cause alterations in mood (Penton-Voak, 2013). Facial expressions influence the behavior of the observer through mirroring or contagion and allow for more fluent and successful social interactions (Frith, 2009). Since the spread of COVID-19, face masks have become mandatory in countries around the globe. Face masks are successful in preventing the spread of COVID-19 (Liang et al., 2020); however, face masks have the potential for creating errors in facial processing which could lead to miscommunication or attribution errors. Previous research finds that facial ambiguity and occlusion lead to more errors in facial perception (Bassili, 1979; Blais et al., 2012; Carbon, 2020; Grundmann et al., 2021; Kasterndieck et al., 2021; Kret & de Gelder, 2012; Roberson et al., 2012). As such, individuals who are less adept at social interactions may be disadvantaged when interacting with others who are wearing face masks.

Research on the effects of face occlusion on emotion perception has taken artificial (e.g., occluding with bubbles, Blais et al., 2012; rectangles, Bassili, 1979; or ovals, Roberson et al., 2012) and ecologically valid (e.g., occluding with scarves, Kret & de Gelder, 2012; sunglasses, Roberson et al., 2012; or face masks, Carbon, 2020; Freud et al., 2020; Grundmann et al., 2021; Kastendieck et al., 2021) approaches. In general, it is found that occluding parts of the face reduce the perceptional accuracy of emotion expressions. There is support for some emotion expressions being more negatively impacted than others. For example, the lower portion of the face, including the mouth, has been found to be more important for accurately identifying happiness and sadness, while the upper portion of the face, including the eyes, has been found to be more important for accurately identifying anger and fear (Bombardi et al., 2013).

The recent increase in the global use of face masks has sparked interest in the implications for emotion identification. Carbon (2020) had participants identify the emotional expression (angry, disgusted, fearful, happy, neutral, and sad) of faces presented with and without face masks. Face masks reduced accurate perception of emotion expression for all emotions except for fearful and neutral expressions. Further, participants reported less confidence in their perception of emotion expression when making judgments about faces presented with face masks. It was also found that angry and disgusted were frequently misattributed with each other, while neutral was frequently misattributed for happy and sad.

In a similar study, Grundmann and colleagues (2021) presented participants with faces with and without face masks and had them make emotion expression judgments from the same expressions as Carbon (2020). Again, perception accuracy for emotion expressions presented with face masks was significantly decreased compared to emotion expressions presented without face masks. This effect was significantly larger for older participants, male participants, and when making a judgment about an older face presented with a face mask. Interestingly, participants were more likely to provide lower closeness ratings for masked versus unmasked faces overall, but also rated masked negative faces as being more trustworthy, likable, and close than unmasked negative faces, supporting the emotion-as-social-information model (Kleef, 2009).

Kastendieck and colleagues (2021) added to the ecological validity of face mask studies by using a dynamic emotion database and face tracking to add face masks to their stimuli. These were short videos of happy and sad expressions. The dynamic information seemed to increase emotion perception accuracy, as it was at ceiling in their task; however, faces presented with face masks were still found to decrease intensity ratings of the expressions and ratings of interpersonal closeness. Facial mimicry was also measured in this study. Face masks were found to significantly reduce the amount of mimicry of happy expressions in the perceiver but not sad expressions, further supporting the finding that the communication of happy expressions may be particularly impacted by face mask use.

While Grundmann and colleagues (2021) found that age and gender were associated with accurate perception of emotion expression on faces, other individual differences associated with perceiving emotion in masked faces have not been explored. One possible indicator of face perception accuracy is social intelligence. Social intelligence refers to the ability to understand the abilities of others, the social context, and the perspective of others, as well as predict their reactions (Silvera et al., 2001). Populations associated with lower social intelligence, such as those diagnosed on the autism spectrum, show impaired perception of faces and subsequently atypical social communication (for a review, see Webb et al., 2017).

The Reading the Mind in the Eyes (RMET) test (Baron-Cohen et al., 1997) was originally developed to test differences in social intelligence between normal functioning adults and adults with Asperger syndrome or high-functioning autism. The test was updated in 2001 (Baron-Cohen et al., 2001) to detect the subtle and subjective differences in social intelligence between normal functioning adults. In this test, participants are presented with images of just the eyes from various faces. Each image is accompanied by four unique words, and participants are asked to select the one that best represents what the person is thinking or feeling. The RMET test is described as an advanced theory of mind test. Theory of mind is the ability to attribute mental states of oneself or another person. The RMET has been found to predict emotional intelligence (Megías-Robles et al., 2020). Thus, the RMET test could potentially predict one’s ability to identify emotions, even if the emotional expression is not completely clear, such as when someone is wearing a face mask.

Another way to assess social intelligence is through established self-report measures. The Tromsø Social Intelligence scale (TSIS) is a self-report measure that tests for three factors of social intelligence: social information processing, social skills, and social awareness (Silvera et al., 2001). The components of social intelligence in the TSIS incorporate the perception of internal states, dealing with people, knowledge about social and life rules, insight and sensitivity in complex situations, social techniques, perspective taking, and social adaptability. Theoretically, individuals with higher levels of social intelligence should have better perception of masked faces and, as such, have more successful social interactions and communication during the COVID-19 pandemic.

The goals of the present study were to investigate the effects of face masks on the perception of emotion expressions, the types of emotions misattributed to incorrectly perceived expressions with and without face masks, and the ability of measures of social intelligence to predict emotion perception accuracy. We had several hypotheses for this study design: Hypothesis 1: face masks will significantly decrease emotion perception accuracy, Hypothesis 2: accurate perception of emotion expressions that rely more on features of the mouth, such as happy, disgust and fear, will be more negatively impacted by face masks than expressions that rely more on the eyes, such as angry and sad, Hypothesis 3: angry and disgust will be misattributed with each other, and Hypothesis 4: individual differences in social intelligence, as measured by performance on the RMET and scores on the TSIS, will predict emotion perception accuracy.

## Methods

### Participants

Participants were recruited via postings on Facebook and through an introductory psychology participant pool. Participants recruited via Facebook were volunteer participants and were not compensated for their participation. The IP addresses for participants recruited via Facebook were restricted to the USA. Participants recruited via the participant pool were students enrolled in introduction to psychology courses at Weber State University in Ogden, Utah, and received course credit as compensation for their participation.

Of the 259 total participants, 35 participants had incomplete data and were removed from the study, resulting in a final sample size of 224. Age of the participants ranged from 18 to 64 years (*M* = 23.5, *SD* = 8.0). All participants in the sample reported as either male (*N* = 88), female (*N* = 133), non-binary/third gender (*N* = 2) or prefer not to say (*N* = 1). Ethnicity was also reported (1.8% African American/Black, 1.8% Asian, 84.4% Caucasian/White, 8.5% Hispanic/Latinx, 8.4% Native American, 2.2% Other, 0.9% prefer not to say).

### Materials

#### Reading the mind in the eyes test

The Reading the Mind in the Eyes test (RMET; Baron-Cohen et al., 2001) consists of 37 images depicting the eyes of individuals’ faces. These images originate from British magazines from the 1990s. Each image had four non-repeating emotional words that accompanied it (e.g., puzzled, nervous, insisting, contemplative for one image and ashamed, nervous, suspicious, indecisive for another image). The RMET has been used as a measure of social intelligence (Baron-Cohen et al., 2001; Megías-Robles et al., 2020).

#### Face stimuli

**Fig. 1:**
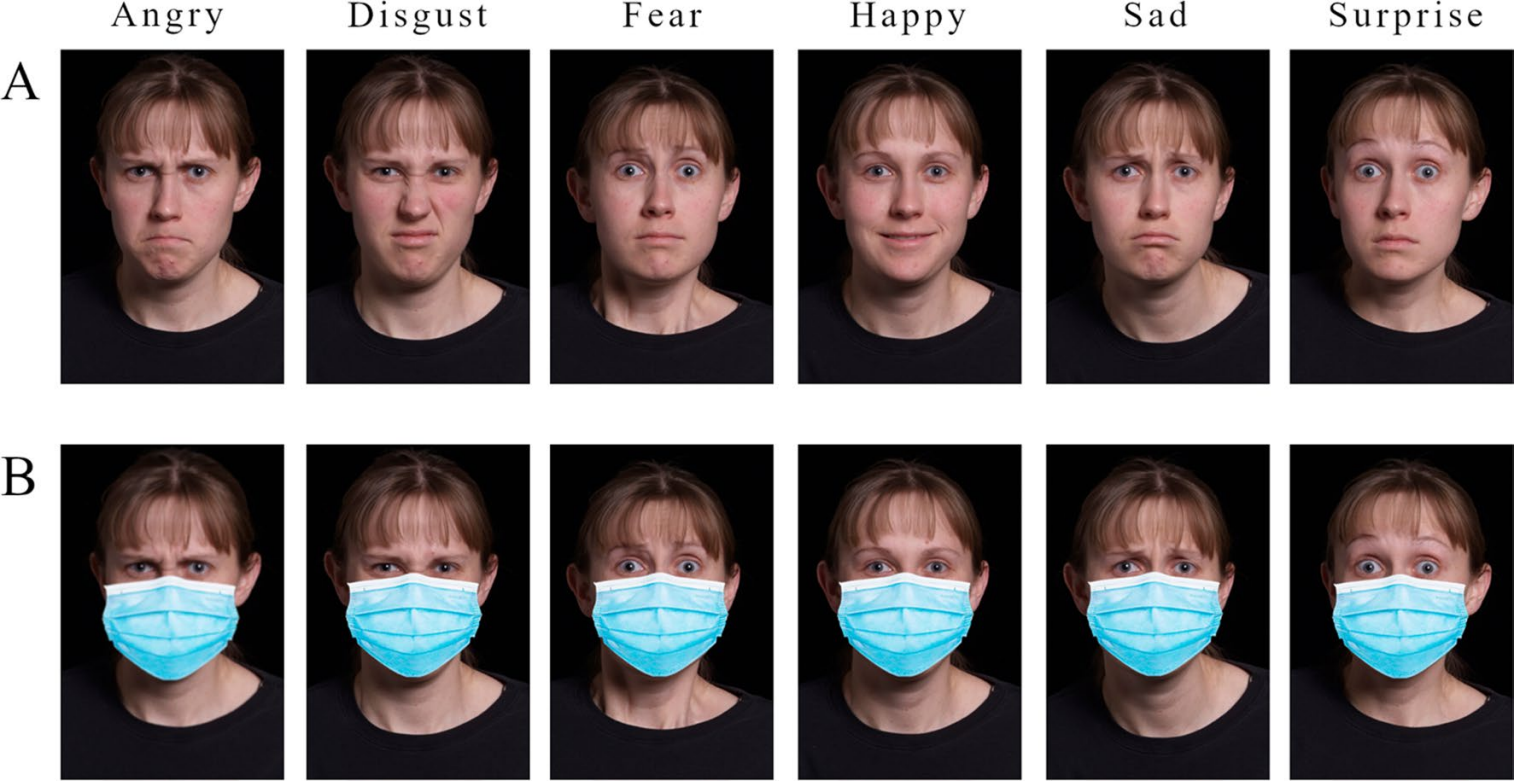
Sample of images used for masked and unmasked conditions. **A** A person showing six different emotions without a mask. **B** The same person showing six different emotions with a mask

For the Emotion Perception Task, face stimuli of two male individuals and two female individuals from the IASLab Face Set (see https://www.affective-science.org/face-set.shtml) were used. Six facial expressions: fear, anger, surprise, happy, sad, and disgust were used for each individual, resulting in 24 face stimuli. Adobe Photoshop 2021 version 22.x was used to impose a blue surgical mask on to each of the face stimuli resulting in 48 total stimuli: 24 with face masks and 24 without face masks (Fig. 1).

The IASLab Face Set has each face stimulus centered in its frame by the eyes such that the midpoint between the eyes is centered on the image, and all the eyes are at the same height of the image frame. Blue, surgical face masks were added to the face stimuli by centering the top of the nose covering portion of the mask on the image 40% down from the top of the image frame. As such, the location of the face mask was uniform across all stimuli.

#### Tromsø social intelligence scale

The Tromsø Social Intelligence Scale (TSIS) includes 21 items assessed on a 7-point Likert scale ranging from *describes me extremely poorly* to *describes me extremely well.* The TSIS has three subscales: Social Information Processing, Social Skills, and Social Awareness. Items assess social awareness and behaviors, for example: “*I can predict other peoples’ behavior”* and “*I have a hard time getting along with other people”* (Silvera et al., 2001).

### Procedure

The study was conducted via Qualtrics. First, digital informed consent was obtained. Following, participants performed three tasks: the Reading the Mind in the Eyes task, the Emotion Perception Task, and the TSIS. Time of response was not recorded, but participants were prompted to answer each question as quickly and accurately as possible.

Presentation of the stimuli for the RMET was randomized across individuals. The purpose of randomization was to prevent priming effects between images. Participants were asked to answer as quickly as possible by selecting the word they felt best represented the emotion expressed. There were 37 total RMET trials. For the original RMET task see Baron-Cohen and colleagues (2001).

Following completion of the RMET, participants completed the Emotion Perception Task. Presentation of the stimuli for the Emotion Perception Task was also randomized between participants. Participants were asked to answer as quickly as possible by selecting the label they felt best represented the emotion expressed. For the Emotion Perception Task, each of the face stimuli was presented with the same six label options: fear, anger, surprise, happy, sad, and disgust. There were 48 total Emotion Perception trials (24 with face masks, 24 without face masks). For each of the emotional expressions, two were presented with face masks, and two without face masks, and for each of those, one female and one male face was presented.

Following the Emotion Perception Task, the TSIS was completed, and questions about mask attitudes and demographic questions were completed. Once all questions were answered, participants were thanked for their participation and exited the Qualtrics platform. Data were exported to R v.4.1.2 for the linear mixed model analysis, and lme4, lmerTest, r2glmm, multilevelTools, and mitml libraries were used. SPSS v.25 was utilized for the chi-square analyses.

## Results

### Emotion perception task

Overall accuracy on the Emotion Perception Task was *M* = 0.70 (*SD* = 0.08). Accuracy on the Emotion Perception Task was modeled as a function of Mask and Emotion as crossed fixed effects, with RMET accuracy and TSIS scores entered as fixed covariates and participant as a cluster variable, for which a random effect was estimated. Prior to analysis, we effect-coded Mask (no face mask = 0, face mask = 1) and Emotion (anger = 3, disgust = 2, fear = 1, sad = − 1, happy = − 2, surprise = − 3) and grand mean centered RMET accuracy and TSIS scores. A 2-level multilevel model was used to account for accuracy on the Emotion Perception Task with random slopes estimated for Mask conditions for each participant, and random intercepts for RMET accuracy and TSIS score were estimated for each participant. Estimates were made using an unstructured covariance matrix and Satterthwaite approximation method of estimating degrees of freedom. The intraclass correlation coefficient was small, ρ = 0.12, *df* = 222, *p* = 0.07, suggesting that accuracy across levels of Mask and Emotion was not fully independent within the same participant and confirming that a multilevel analysis was possible for these data.

Effect sizes were estimated with semi-partial R^2^ (R_β_^2^, Edwards et al., 2008). There was a main effect of Mask on accuracy, *b* = -0.0.52, *MSE* = 29.77, *F*(1, 2451.49) = 768.87, *p* < 0.001, *R*_*β*_^*2*^ = 0.25, such that accuracy was lower on face mask trials (*M* = 0.63, *SD* = 0.09) than no face mask trials (*M* = 0.78, *SD* = 0.09). RMET accuracy also significantly predicted Emotion Perception Task accuracy, *b* = 0.23, *MSE* = 1.33, *F*(1, 221.73) = 34.39, *p* < 0.001, *R*_*β*_^*2*^ = 0.23, such that increases in RMET accuracy predicted increases in Emotion Perception Task accuracy. TSIS scores were not found to significantly predict accuracy, *MSE* = 0.03, *F*(1, 221.73) = 0.86, *p* = 0.35, *R*_*β*_^*2*^ = 0.003. There was also a significant interaction between Mask and Emotion, such that Emotion moderated the effect of Mask on accuracy, *MSE* = 15.22, *F*(1, 2453.00) = 393.20, *p* < 0.001, *R*_*β*_^*2*^ = 0.13. RMET accuracy nor TSIS scores were found to significantly moderate this interaction.

**Fig. 2:**
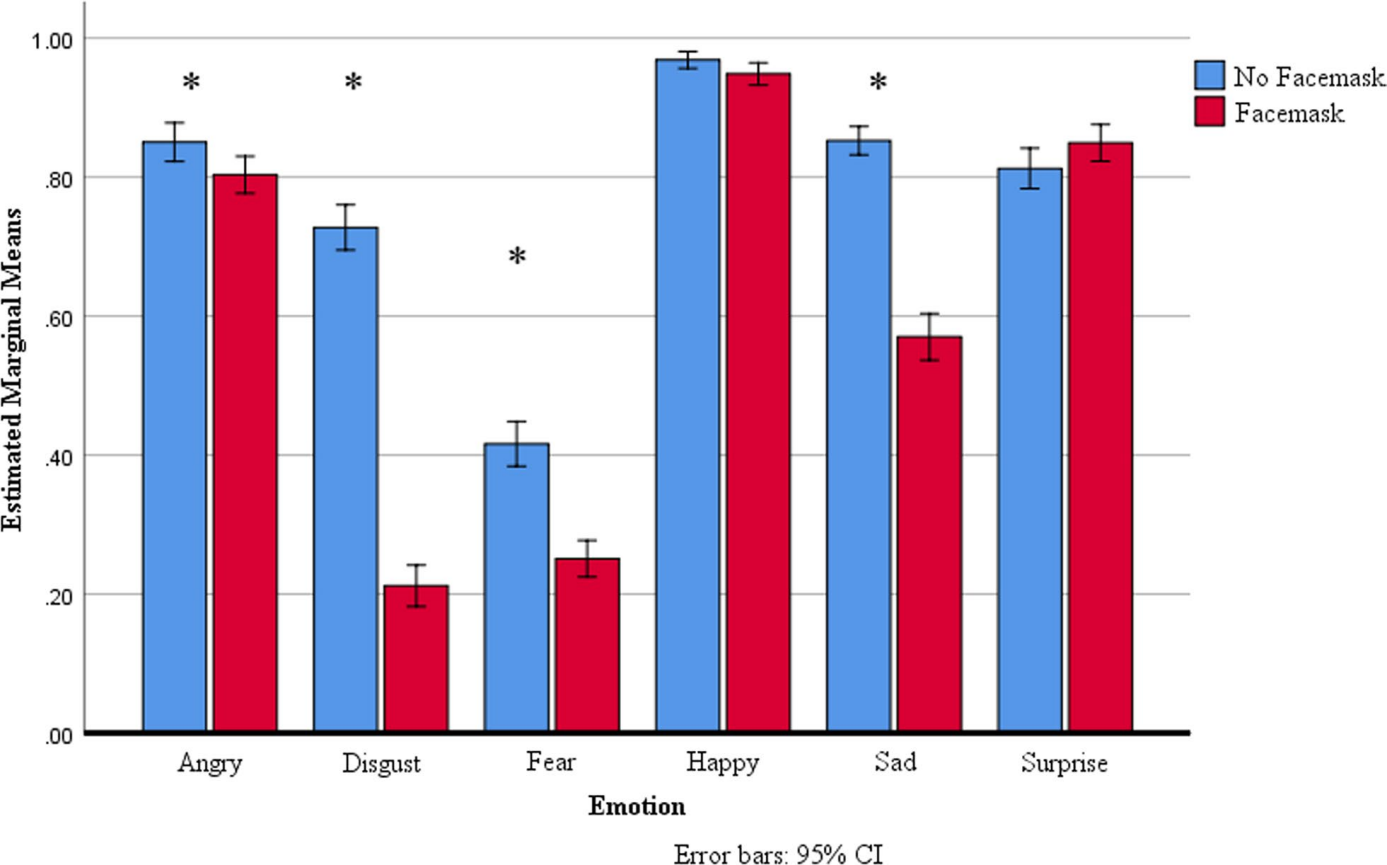
Mean accuracy of response for Emotion in face mask and no face mask conditions. Mean percentage of correct assessment of the emotional states for faces with masks (red) and faces with no mask (blue). Error bars indicate 95% confidence intervals. Asterisks indicate statistical difference with Bonferroni-corrected alpha level (*α* < .008)

To break down the Mask and Emotion interaction, Bonferroni-corrected t-tests were used to compare accuracy across levels of Mask for each level of Emotion (Fig. 2). When the target face displayed anger, accuracy on no face mask trials (*M* = 0.85, SD = 0.21) was significantly higher than accuracy on face mask trials (*M* = 0.80, SD = 0.20), with a mean difference of 0.05, *t*(223) = 2.93, *p* = 0.004, 95% *CI*[0.02, 0.08], *d* = 0.24. When the target face displayed disgust, accuracy on no face mask trials (*M* = 0.73, *SD* = 0.25) was significantly higher than accuracy on face mask trials (*M* = 0.21, *SD* = 0.23), with a mean difference of 0.52, *t*(223) = 26.30, *p* < 0.001, 95% *CI*[0.48, 0.55], *d* = 2.16. When the target face displayed fear, accuracy on no face mask trials (*M* = 0.42, *SD* = 0.24) was significantly higher than accuracy on face mask trials (*M* = 0.25, *SD* = 0.20), with a mean difference of 0.17, *t*(223) = 9.02, *p* < 0.001, 95% *CI*[0.13, 0.20], *d* = 0.77. When the target face displayed sadness, accuracy on no face mask trials (*M* = 0.85, *SD* = 0.16) was significantly higher than accuracy on face mask trials (*M* = 0.57, *SD* = 0.26), with a mean difference of 0.28, *t*(223) = 15.68, *p* < 0.001, 95% *CI*[0.25, 0.32], *d* = 1.30. When the target face displayed happy or surprise, no significant difference was found between no face mask and face mask trials (happy: *t*(223) = 2.05, *p* = 0.04, no face mask *M* = 0.97, *SD* = 0.09, face mask *M* = 0.95, *SD* = 0.12; surprise: *t*(223) = -2.26, *p* = 0.03, no face mask *M* = 0.81, *SD* = 0.22, face mask *M* = 0.85, *SD* = 0.20).

### Emotion misattribution

**Fig. 3:**
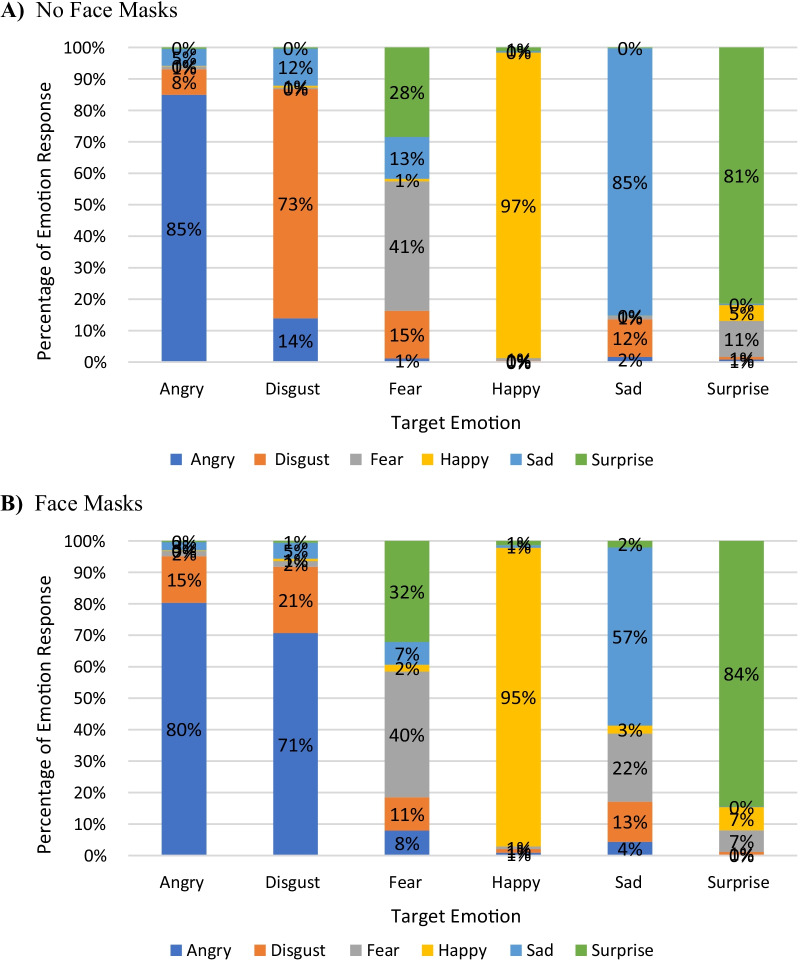
Percentage of each emotion selected for each emotion condition. Each bar graph represents the mean percentage of correct and incorrect responses for no face mask (**A**) and face mask (**B**) conditions. Figure **A** shows the total correct responses in angry faces is 85%, while only 8% misattributed angry faces as disgust for unmasked conditions. Participants correctly identified disgust faces 71% in unmasked conditions while misattributing disgust with anger 21% of the time. Figure **B** shows the total correct responses in angry faces are 80%, while only 15% misattributed angry faces as disgust for masked conditions. Participants, however, correctly identified disgust faces in masked conditions only 21% of the time while misattributing digust faces for angry faces 71% of the time

To investigate which expressions were the most commonly misattributed, a Pearson Chi-square test was conducted between Mask conditions for incorrect trials only for each target emotion face. For incorrect responses to angry faces, disgust was the most common misattributed emotion for both face mask (74.0%) and no face mask (54.4%) trials, *χ*^*2*^(4, *N* = 371) = 20.64, *p* < 0.001, with a weak to moderate association (*V* = 0.26). For incorrect responses to disgust faces, anger was the most common misattributed emotion for both face mask (89.3%) and no face mask (50.6%) trials, *χ*^*2*^(4, *N* = 985) = 205.17, *p* < 0.001, with a relatively strong association (*V* = 0.46). For incorrect responses to fear faces, surprise was the most common misattributed emotion for both face mask (53.0%) and no face mask (49.4%) trials, *χ*^*2*^(4, *N* = 1111) = 77.37, *p* < 0.001, with a weak to moderate association (*V* = 0.26). For incorrect responses to sad faces, fear was the most common misattributed emotion for face mask trials (50.4%), and disgust was the most common misattributed emotion for no face mask trials (78.7%), *χ*^*2*^(4, *N* = 533) = 15.23, *p* < 0.001, with a relatively strong association (*V* = 0.46). For incorrect responses to surprise faces, fear was the most common misattributed emotion for both face mask (45.5%) and no face mask (62.8%) trials, *χ*^*2*^(4, *N* = 315) = 15.23, *p* = 0.004, with a weak to moderate association (*V* = 0.22). For incorrect responses to happy faces, there was not a most common misattributed emotion, *χ*^*2*^(4, *N* = 75) = 6.64, *p* = 0.16, as accuracy on both no face mask and face mask trials for happy faces was at ceiling. See Fig. 3 for percent of each misattributed emotion for each target face.

## Discussion

The COVID-19 pandemic has affected social interactions in a myriad of ways. One of the most notable changes is the required use of face masks in most public settings. While this is necessary for public health and safety, interacting with others while wearing face masks can impact our perception of those interactions. In this study, participants indicated the emotional expression perceived on faces with and without face masks. In support of Hypothesis 1 and replicating previous research (Carbon, 2020; Grundmann et al., 2021; Kastendieck et al., 2021), emotion perception accuracy was significantly decreased when faces were presented with face masks. In support of Hypothesis 2, perception of some emotion expressions was more negatively impacted by face masks than others, however, not in the expected direction. Despite the expression of happiness relying more on features of the mouth (Bombardi et al., 2013), both happy and surprise faces were perceived equally well (near ceiling) when presented with or without a face mask. Instead, very large negative effects of face masks were found on perception of disgust and sad faces, and the effects were also large for the perception of fear faces and small for angry faces. There was support for Hypothesis 3, as the most common misattributed emotion for the expression of disgust was angry and vice versa. Misattributed emotions for other expressions were varied. Finally, Hypothesis 4 was also supported. Individual differences in social intelligence, as measured by the RMET test, did predict perception accuracy, however, scores on the TSIS did not.

Facial emotion recognition accuracy relies on facial features with high diagnostic values. Facial action units show sad, fearful, and angry faces rely on upper facial features, while happy and disgust rely on lower facial features (Wegrzyn et al., 2017a, b). Research on the effects of covering the lower half of the face on perceiving negative vs. positive emotions has been mixed. One study found that covering lower facial features results in less accurate perception of positive emotions and more intense interpretation of negative emotions (Fischer et al., 2011). Another found that covering lower facial features results in more socially desirable perceptions of negative expressions (Grundmann et al., 2021). We found perception accuracy for happy expressions to be the highest in both face mask and no face mask conditions, so perception of this expression was not affected by covering the mouth. For other expressions, we found angry and disgust to be most commonly misattributed with each other, as well as fear and surprise. Sad was the only expression to have different misattributions when presented with a face mask (fear) versus without a face mask (disgust). This confusion among other expressions likely contributes to increased perceptions of generally negative expressions, consistent with other research (Carbon 2020; Fischer et al., 2011; Wegrzyn et al., 2017a,  b).

While overall accuracy on the Face Perception Task and the RMET test was comparable, accuracy on the RMET test was higher than accuracy on Face Perception Task trials with a face mask. This is of interest because the RMET provides less face expression information (i.e., just an image of the eye portion of a face) than a masked face. Perception of masked faces may be further distorted due to the sociocultural associations of face mask use and the COVID-19 pandemic. Americans have reported an increase in poor social interactions and the amount of anxiety-related symptoms they experience since the start of the pandemic (Czeisler et al., 2020; Ettman et al., 2020; Taquet et al., 2020). Some of the reported stressors include social isolation and an inability to predict future pandemic outcomes (Shah et al., 2020). These lower levels of social connectedness (Neta & Brock, 2021) and higher degrees of social anxiety (Maoz et al., 2016) have a propensity to increase sensitivity to negative affect. Furthermore, the very nature of the face mask could make a difference in how one perceives emotions of others. Kret and Fischer (2017) found that participants were more likely to associate negative affect depending on the type of facial coverings. They found participants to rate faces as more positive when covered with a Western headdress compared to an Islamic headdress and were more likely to identify faces with an Islamic headdress as sad. Individual negative and positive biases toward face mask use may also be skewing the perception of faces with face masks.

The RMET test was a reliable predictor of emotion perception accuracy regardless of the presence of face masks. When looking at the relationship between RMET performance and emotion perception performance, significant relationships were not found for the perception of happy and disgust expressions. Happy was the most accurately perceived expression, and disgust was the least accurately perceived expression, which suggests that the type of social intelligence assessed by the RMET test does not help with discriminating extremely identifiable or not identifiable expressions. In contrast, the participant’s performance during the RMET task was able to predict their ability to correctly perceive anger, sadness, and surprise facial expressions in face mask and no face mask conditions. To our knowledge, these results represent the first direct demonstration of using social intelligence to predict emotion-recognition abilities of individuals wearing face masks.

### Limitations

The data collected for this study were all collected online via Qualtrics. Certain controls expected from laboratory experiments were not present. Participants were allowed to take the survey from any location in the USA with internet access, and thus, screen resolution, computer hardware, and imaging contrasts were beyond our control. The study did encourage participants to answer questions as fast as they could, but we could not collect reaction time data. Perhaps collecting reaction time in future studies could help determine if there are biases to processing speeds for masked versus unmasked faces regardless of accuracy.

Another limitation of this study was the order in which tasks were administered. We randomized the order within tasks but did not randomize the order between them. This means that the RMET test was always first, the Emotion Perception Task was presented second, and the TSIS was last. It could be the case that during the Emotion Perception Task, participants were either getting bored with identifying faces or were perhaps getting even better at it, after having completed the RMET test. We might see different results if we randomized the order of the tasks.

### Implications and future research

Our study found that face masks impair emotion perception accuracy, and social intelligence can predict that accuracy even when someone is wearing a face mask. Recent studies have shown that social intelligence is a skill that can improve with practice (Geßler et al., 2020). We were surprised to not find a relationship between the TSIS and emotion perception accuracy. The RMET has also been found to predict emotional intelligence (Megías-Robles et al., 2020), as such, we suggest that future research should also assess emotional intelligence as a possible underlying individual difference driving this effect.

The risk factors associated with wearing a face mask are minimal in comparison to the consequences of not wearing a face mask in the midst of a pandemic. Face masks are an effective strategy in preventing the spread of COVID-19 and other respiratory-related viruses (Liang et al., 2020). As such, it is important to raise awareness of the potential consequences of wearing face masks to successful social interactions and subsequent mental health. In particular, individuals who struggle with perceiving nonverbal social and emotional cues from faces in general are going to be even more socially disadvantaged when communicating with individuals who are wearing face masks. Patience and extra care toward ensuring that intentions are communicated clearly while interacting with others while wearing face masks are needed.

## Data Availability

The datasets used and/or analyzed during the current study are available from the corresponding author on reasonable request.
